# Three-dimensional anatomy of equine incisors: tooth length, enamel cover and age related changes

**DOI:** 10.1186/1746-6148-9-249

**Published:** 2013-12-09

**Authors:** Patricia Schrock, Matthias Lüpke, Hermann Seifert, Carsten Staszyk

**Affiliations:** 1 Institute of Anatomy, University of Veterinary Medicine Hannover, Bischofsholer Damm 15, D-30173 Hannover, Germany; 2 Institute for General Radiology and Medical Physics, University of Veterinary Medicine Hannover, Bischofsholer Damm 15, D-30173 Hannover, Germany; 3 Institute for Veterinary-Anatomy, -Histology and -Embryology, Faculty for Veterinary Medicine, Justus-Liebig-University Giessen, Frankfurter Str. 98, D-35392 Giessen, Germany (formerly Institute of Anatomy, University of Veterinary Medicine Hannover, Bischofsholer Damm 15, D-30173 Hannover, Germany

**Keywords:** Tooth, Horse, Enamel, Dentin, Incisor, EOTRH

## Abstract

**Background:**

Equine incisors are subjected to continuous occlusal wear causing multiple, age related changes of the extragingival crown. It is assumed that the occlusal wear is compensated by continued tooth elongation at the apical ends of the teeth. In this study, μCT-datasets offered the opportunity to analyze the three-dimensional appearance of the extra- and intraalveolar parts of the enamel containing dental crown as well as of the enamel-free dental root. Multiple morphometric measurements elucidated age related, morphological changes within the intraalveolar part of the incisors.

**Results:**

Equine incisors possess a unique enamel cover displaying large indentations on the mesial and distal sides. After eruption tooth elongation at the apical end outbalances occlusal wear for two to four years resulting in increasing incisor length in this period of time. Remarkably, this maximum length is maintained for about ten years, up to a tooth age of 13 to 15 years post eruption. Variances in the total length of individual teeth are related to different Triadan positions (central-, middle- and corner incisors) as well as to the upper and lower arcades.

**Conclusion:**

Equine incisors are able to fully compensate occlusal wear for a limited period of time. However, after this ability ceases, it is expected that a diminished intraalveolar tooth length will cause massive changes in periodontal biomechanics. The time point of these morphodynamic and biomechanical changes (13 to 15 years post eruption) occurs in coincidence with the onset of a recently described destructive disease of equine incisor (equine odontoclastic tooth resorption and hypercementosis) in aged horses. However, further biomechanical, cell biological and microbiological investigations are needed to elucidate a correlation between age related changes of incisor morphology and this disease.

## Background

Equine incisors had been often investigated addressing the visible aspects of the teeth, i.e. the clinical crown and the occlusal surface. Several studies examined morphological characteristics of the clinical crown (i.e. shape of the occlusal surface, disappearance of infundibula, occurrence of dental stars, appearance and position of the Galvayne’s groove and occurrence of upper corner incisor hooks) in order to find correlations between certain anatomical features and the age of the horse [1-4]. Remarkably wide variations and breed specific characteristics were demonstrated indicating that aging of horses by their incisor dentition is very vague [2]. Other investigations focused on the histological and ultra-structural description of the dental hard substances of the occlusal surface of the equine incisors [5-11]. Those studies demonstrated that equine specific formations of primary, secondary and tertiary dentin guarantee an effective dynamic seal of the pulp although the tooth is continuously subjected to massive occlusal wear.

The complex and overlapping arrangement of incisors within the incisor arcade hamper the description of the three-dimensional positions of individual teeth, especially while evaluating 2D radiographs. Furthermore, the enormous length of equine incisors (more than 80 mm in young horses) hamper the histological identification of dental hard substances along the complete tooth length. Therefore, anatomical data of incisors including extra- and intra alveolar parts of the teeth are very rare [3]. Meanwhile, modern techniques like micro-computed tomography (μCT) allow to overcome the mentioned limitations. By applying appropriate techniques and protocols the three dimensional arrangement of the incisor arcade as well as the composition of hard substances can be investigated not only in tooth sections, but in complete teeth. The need for these data largely arises from clinical observations concerning equine specific dental disorders.

Recently, a painful and progressive incisor disease named Equine Odontoclastic Tooth Resorption and Hypercementosis (EOTRH) gained much attention in the field of equine dentistry [12]. Remarkably, EOTRH is a disease of aged horses with most animals being older than 12 years when first signs are diagnosed. The predominant symptoms (odontoclastic tooth resorption and hypercementosis) are not uniform distributed along the teeth but affect the palatal/lingual aspects of the intraalveolar part of the tooth. There is an obvious correlation of the disease with the tooth age and with distinct anatomical areas of the intraalveolar part of the tooth. Therefore we intended to investigate the morphometric and morphologic appearance of equine incisors and their age related alterations. Using modern high resolution μCT we aimed to examine the three-dimensional appearance of the teeth as well as their individual composition of dental hard substances.

## Methods

### Material

Heads from 14 horses were obtained in the clinic for horses at the University of Veterinary Medicine Hannover, after euthanasia for other than dental reasons. All specimens were obtained from horses euthanized for other reasons than for this study. The heads were deep frozen and stored until further preparations. The age of 12 horses was determined by means of the equine ID card; the age of two horses was estimated using the aging guides by Muylle 1996 and 1999 [2,3] and Martin 2007 [4]. Age, breed and sex of the horses are listed in Table 1.

**Table 1:** Distribution of age, breed and sex

**Horse**	**Age**	**Sex**	**Breed**
1	2	Female	Warm blood
2	6	Gelding	Warm blood
3	6	Gelding	Warm blood
4	7	Gelding	Warm blood
5	7,5	Gelding	Thoroughbred
6	8	Gelding	Warm blood
7	10	Gelding	Warm blood
8	12	Gemale	Haflinger
9	13	Gemale	Warm blood
10	17,5	Gemale	Warm blood
11	17,5	Gelding	Warm blood
12	18,5	Gemale	Warm blood
13	20	Gelding	Warm blood
14	22	Male	Arab horse

### Preparation of samples for μCT-scans

Mandibular and maxillary incisor arcades including the canines were cut by use of a steel band saw (type K 420, Kolbe GmbH, Elchingen, Germany). The samples were thawed and prepared for the micro-computed tomography (μCT) by removing all soft tissue in order to maximize the quality of the μCT-images.

### Creation of 3-D models

The samples were scanned by a μCT-system (XTremeCT, Scanco Medical AG, Brüttisellen, Switzerland) with an isotropic spatial resolution of 82 μm. The occlusal surfaces of the first incisors were adjusted parallel to the scan direction.

The obtained DICOM (Digital Imaging and Communications in Medicine) datasets were imported in the computer program AMIRA (version 5.4.2, Visualization Sciences Group, Merignac Cedex, France). For each specimen 1000–2000 2D μCT images were created. In order to maximize the picture quality and to optimize the program internal functions of AMIRA, noise reduction filters were used. Subsequently, 3D models of the incisor arcades were constructed. Therefore the different structures (compact bone, periodontal space, enamel, dentin and cement, pulp cavity) with their material specific gray scales were marked on each of the 2D μCT images. Special attention was paid to define detailed interfaces between these structures. For that reason the use of automatic algorithms was limited and most structures were created under visual control. Thereafter 3D surfaces of the materials were generated by the segmented 2D images.

### Morphometric analysis

3D-models of a total of 103 incisors were constructed. The dental age was determined of each tooth and used for further statistical analysis. To enable measurements of different morphological structures, the data sets for each incisor were prepared according to a 2 step procedure:

**Figure 1:**
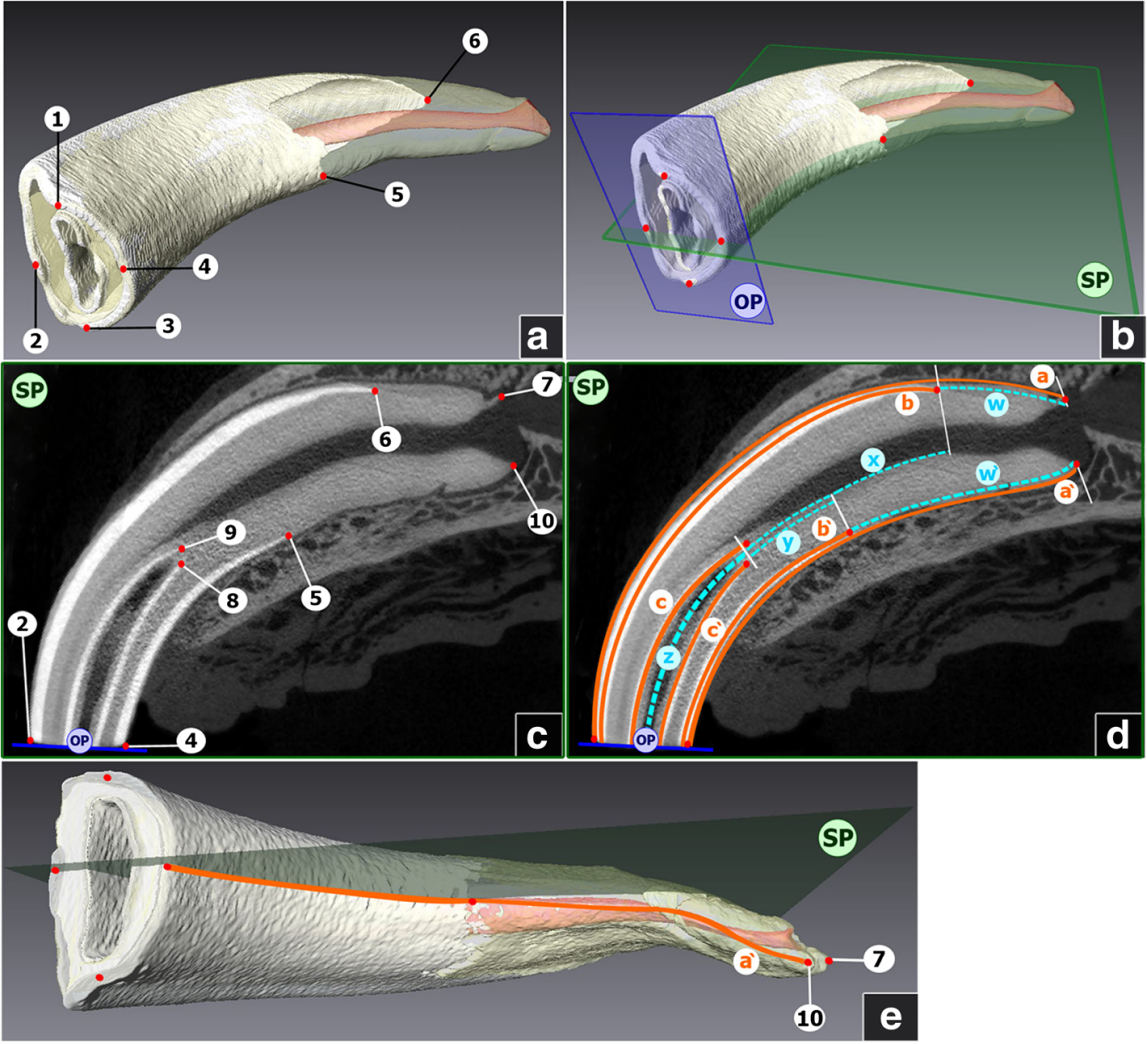
**3D-models and μCT-scans of equine incisors. a**: 3D model of an upper left first incisor (Triadan 201). Numbers indicate reference points (rp). 1 = Most occlusal point of the distal part of the occlusal table; 2 = Most occlusal point of the labial part of the occlusal table; 3 = Most occlusal point of the mesial part of the occlusal table; 4 = Most occlusal point of the palatal part of the occlusal table; 5 = Most apical point of the palatal enamel cover; 6 = Most apical point of the labial enamel cover. **b**: 3D model of an upper left first incisor (Triadan 201). Characters indicate reference planes. OP = Occlusal plane, calculated from rp1-rp4; SP = Sagittal plane, calculated from rp2, rp4, rp5, rp6. **c**: Sagittal μCT - cross section (according to the described sagittal plane SP) of an upper left first incisor (Triadan 201). Characters indicate reference planes. OP = Occlusal plane; SP = Sagittal plane; Numbers indicate reference points (rp). 2 = Most occlusal point of the labial part of the occlusal table; 4 = Most occlusal point of the palatal part of the occlusal table; 5 = Most apical point of the palatal enamel cover; 6 = Most apical point of the labial enamel cover.; 7 = Most apical point of the tooth, labial side; 9 = Most apical point of the infundibular enamel, labial side; 8 = Most apical point of the infundibular enamel, palatal side; 10 = Most apical point of the tooth, palatal side. **d**: Sagittal μCT - cross section of an upper left first incisor (Triadan 201). Lower-case characters and solid orange lines indicate measure-lines; Lower-case characters and turquoise dotted lines indicate calculated lines. a = Labial length of the tooth, a’ = Palatal length of the tooth; b = Labial length of the enamel cover; b’ = Palatal length of the enamel cover; c = Labial length of the infundibular enamel; c’ = Palatal length of the infundibular enamel; w = Labial length of the root; w’ = Palatal length of the root; x = Distance between the apical infundibular limit and the most apical point of the labial enamel cover; y = Distance between the infundibular limit and the most apical point of the palatal enamel cover; z = Mean length of the infundibulum. **e**: 3D model of an upper right first incisor (Triadan 101), view on the palatal side of the tooth. Red points and numbers indicate reference points (rp). 7 = Most apical point of the tooth, labial side; 10 = Most apical point of the tooth, palatal side. Characters indicate reference planes, SP = Sagittal plane. Lower-case character and orange line indicates measure-line. a’ = Palatal length of the tooth. Distances between rps, which are not aligned on the sagittal plane (rp 7 and 10) were measured on the 3D surface, following a virtual plane, bisecting the apex sagittally.

Step 1) Ten anatomical reference points (rp) were identified and marked. The individual reference points are visualized and defined in Figure 1a and 1c.

Step 2) Reference planes were constructed using selected reference points. The position of the occlusal plane (OP) was defined as in-plane with reference points rp1, rp2, rp3 and rp4, analogously the sagittal plane (SP) was defined as in-plane with reference points rp2, rp4, rp5, rp6 (Figure 1b).

### Measurements and calculations

Selected distances were determined, taking the sagittal plane as basis. In the majority of the incisors, reference points 2, 4–10 were aligned on this plane and distances between these points were measured as shown in Figure 1d. In cases, however, in which rp 7 and 10 were not aligned in the sagittal plane, distances a and a’ were measured as shown exemplarily for distance a’ in Figure 1e, using the sagittal plane as basis as well as the 3D surface, following a virtual plane, which bisects the dental root sagittally.

Due to the curved shape of the incisors, distances on the labial and palatal respectively lingual sides were measured separately. For each tooth six distances were measured (Figure 1d and e). Measurements were denoted in mm by use of the so-called B-Spline tool, which is a measuring tool incorporated in the program AMIRA.

Additionally five lengths were calculated (Figure 1d). The mean tooth lengths were calculated as the average lengths of the labial and palatal (lingual) side of the tooth. Analogously the mean length of the enamel cover was calculated. The mean length of the dental root was calculated by subtracting the mean length of the enamel cover (i.e. dental crown) from the mean length of the tooth.

Using the calculated distance between the apical infundibular limit and the apical edges of the enamel cover (as explained in Figure 1d) data was obtained about a post eruptive increase of enamel length.

The relative lengths of the teeth were calculated by dividing the mean length of each tooth by the mean length of all teeth of the same Triadan position. The relative length of the root was calculated as a percentage of the total length of the same tooth.

For bilateral comparison, teeth of eight horses were measured exemplarily on both sides of the jaw. Horse number, age and number of teeth measured in each Triadan quadrant are listed in Table 2.

**Table 2:** Horse number, age, measured Triadan quadrant

**Horse**	**Age**	**Triadan quadrants:**	**1**	**2**	**3**	**4**
		**Number of teeth:**				
1	2*		1			1
2	6		3	3		2
3	6		3	3		3
4	7		3			3
5	7,5		3	3		3
6	8		3			3
7	10		3			3
8	12,5		3	3		3
9	13		3		3	3
10	17,5		3			3
11	17,5		3		3	3
12	18,5		3		3	3
13	20		3			3
14	22		3		3	3
		Σ	40	12	12	39

* = not erupted teeth.

For statistical analysis, Tukey’s Studentized Range Test based on ANOVA (analysis of variance) was used. The Akaike information criterion was used to compare different data fits and to compile trend lines into different diagrams. Statistical analysis was performed with the computer program ORIGIN (version 8.5, OriginLab Corporation, USA).

## Results

### Length of incisors

**Figure 2:**
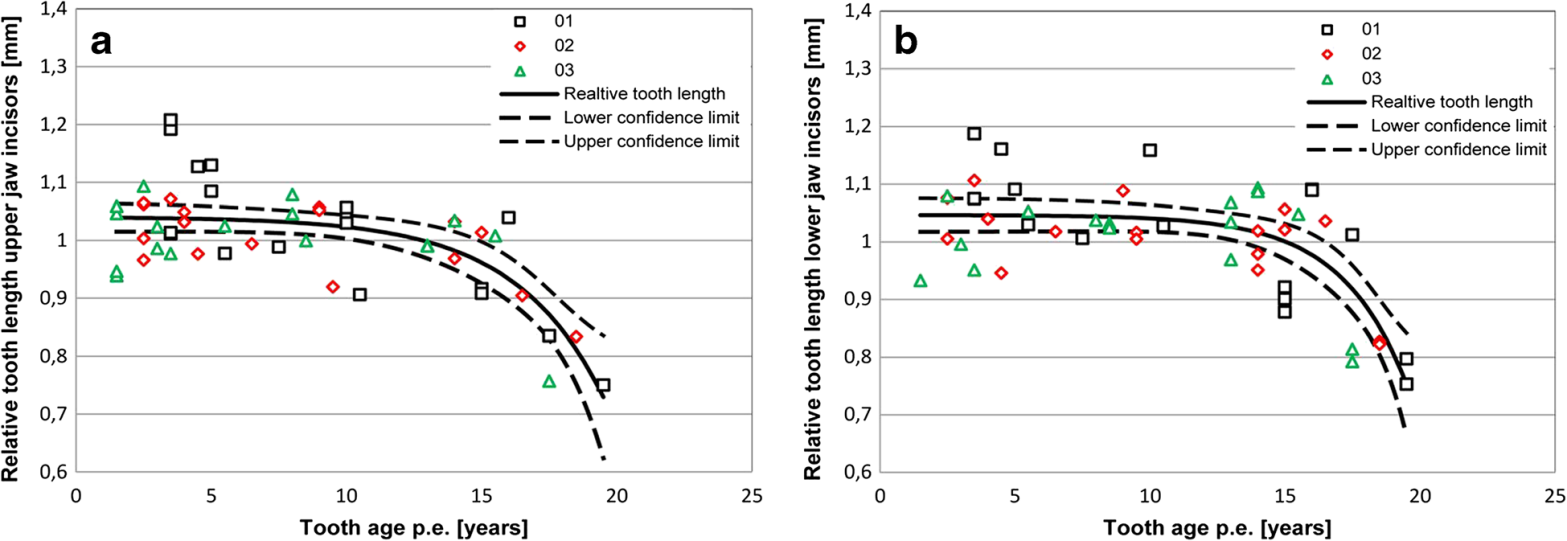
**Relative tooth length of equine incisors. a**: upper jaw incisors. **b**: lower jaw incisors; symbols indicate individual measurements, line indicates trend line and dashed lines indicate upper and lower 95% confidence limit.

**Figure 3:**
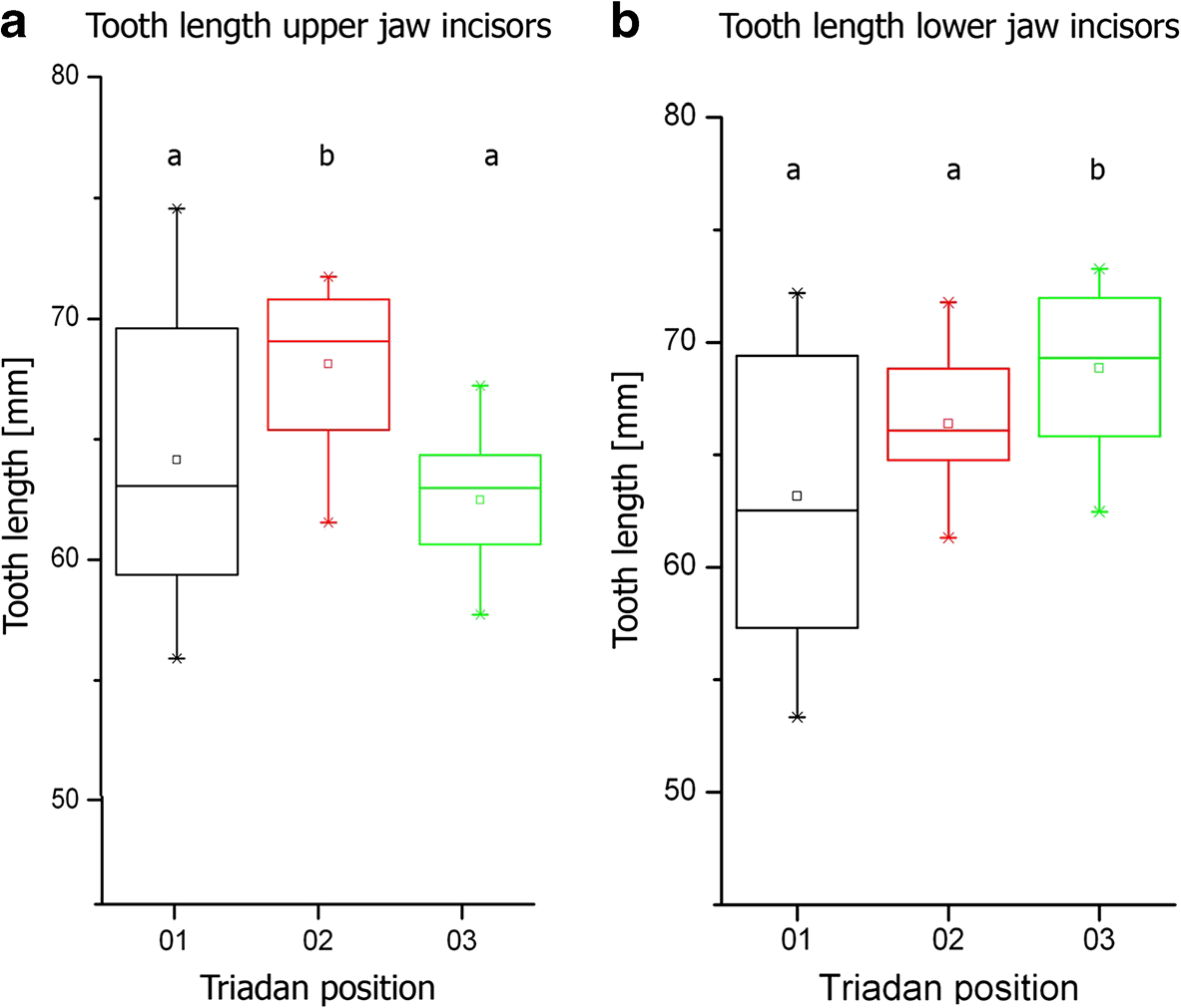
**Boxplots representing the tooth lengths of incisors in different Triadan positions. a**: upper jaw incisors. **b**: lower jaw incisor. Triadan position assigned to the tooth length. Box: interquartile range (25%, 75%), horizontal line: median, center point: mean, whiskers: range (5%, 95%), stars: minimum and maximum. Lower-case characters **(a, b)** indicate a significant difference. (p-values upper jaw incisors: Triadan 01 against 02: p = 0.038, Triadan 01 against 03: p = 0.54, Triadan 02 against 03: p = 0.0019; p-values lower jaw incisors: Triadan 01 against 02: p = 0.16, Triadan 01 against 03: p = 0.007, Triadan 02 against 03: p = 0.32).

Equine incisors reach their maximum length two to four years post eruption (p.e.). This length stays almost constant for about ten years, up to a tooth age of 13 to 15 years p.e., after which the length starts to decrease markedly (Figure 2a and b). There are variations between incisors in different Triadan positions (Figure 3a and b). For comparison teeth in their phase of constant length (i.e. up to an age of 15 years p.e.) were considered only. Upper jaw incisors (Figure 3a) in the middle Triadan position are significant longer than the center (p = 0.038) and corner teeth (p = 0.0019), which show approximately same length. In contrast to this, mandibular corner incisors are significant longer than the center incisors (p = 0.007), whereas the center and middle as well as the corner and middle incisors have the same length.

The lengths of the labial and palatal sides differ in a close range. In the upper jaw center and middle incisors the labial side in mean is about 26% longer than the palatal side. The labial side of the upper corner incisors in mean is 20% longer than the palatal side.

Similar results are seen in lower jaw incisors, with a difference of about 23% between the labial and lingual side in the center and middle incisors and 17% in the corner ones.

As expected, equine incisors display a bilateral symmetry, however small variations between incisors of Triadan quadrants 1 and 2 up to 4.7 mm (8%) and between Triadan quadrants 3 and 4 up to 4.8 mm (9%) were obtained.

### Enamel cover

**Figure 4:**
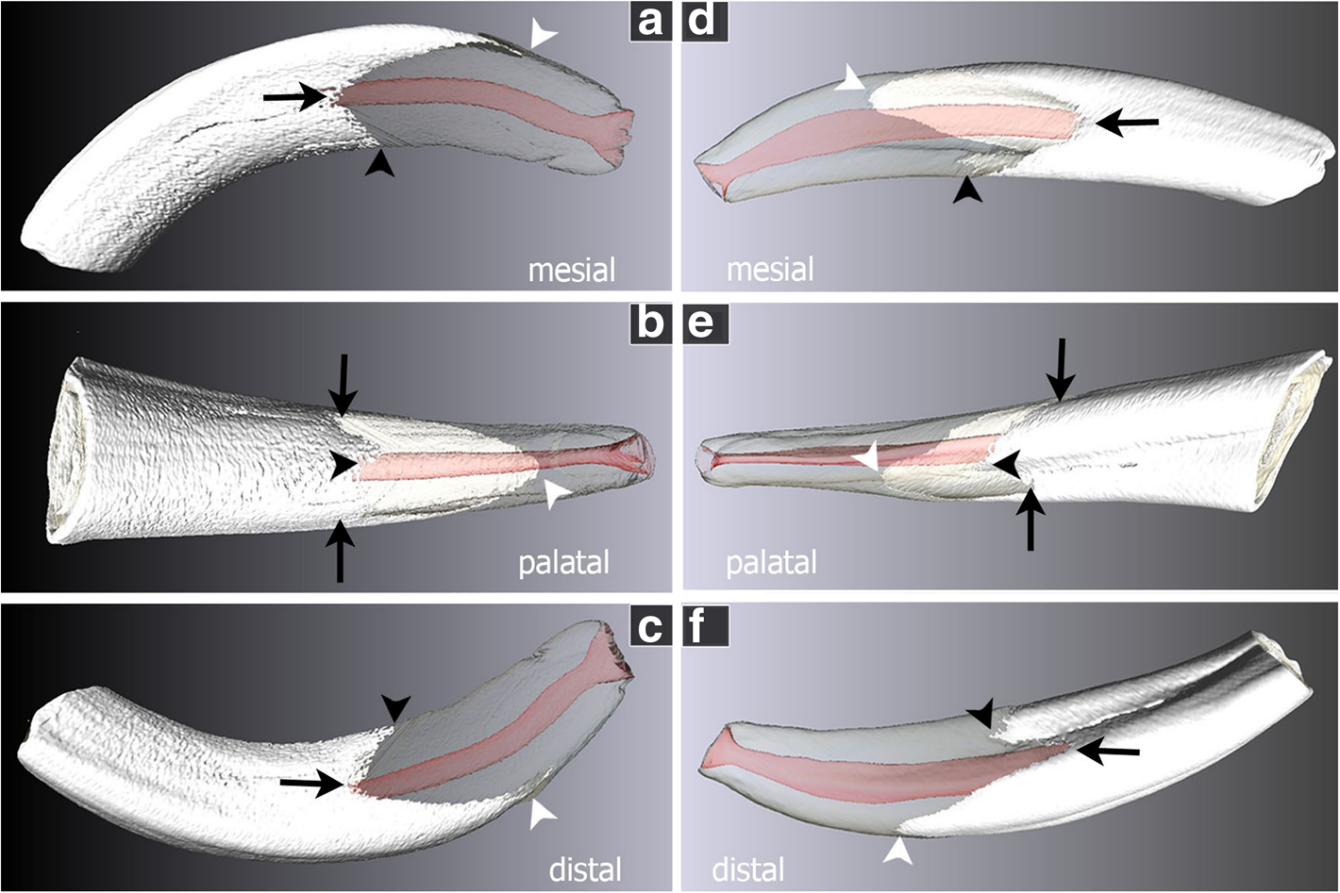
**3D model of incisors, showing the not uniform tube like enamel cover. a**, **b**, **c** Triadan 201 (please also see Additional video file 1) with symmetric indentations (black arrows) and most apical points of the enamel cover (black and white arrow heads) located in the sagittal plane of the tooth. **d**, **e**, **f** Triadan 103 (please also see Additional video file 2) with the mesial incision (**d**, black arrow) reaching further occlusal than the distal one (**f**, black arrow) and the most apical points of the enamel cover located parasagittal (black and white arrow heads). White = enamel cover, transparent = dental root composed of dentin and cementum, red = pulp cavity, black arrows = mesial and distal incision of the enamel cover, black arrow head = most apical point of the palatal enamel cover, white arrow head = most apical point of the labial enamel cover.

The dental crown of incisors consists of three hard substance components: an inner core out of dentine, surrounded by a layer of enamel, which is covered by a thin layer out of cementum. However, the shape of the coronal enamel is not uniform tube like. The enamel reaches labial considerably further apical than palatal respectively lingual (Figure 4a, b, c Additional file 1). On its mesial and distal sides, the coronal enamel shows indentations reaching towards occlusal, leaving the dentin laterally largely uncovered of enamel. These indentations are quite symmetrical in the center incisors in the upper as well as in the lower jaw. In the middle and even more obvious in the corner incisors the mesial indentation reaches clearly further occlusal than the distal one (Figure 4d, e, f Additional file 2). The apical margin of coronal enamel of the center and middle incisors are located near the sagittal plane of the teeth; those of corner incisors however, are shifted distally into a parasagittal plane.

### Length of the dental root

**Figure 5:**
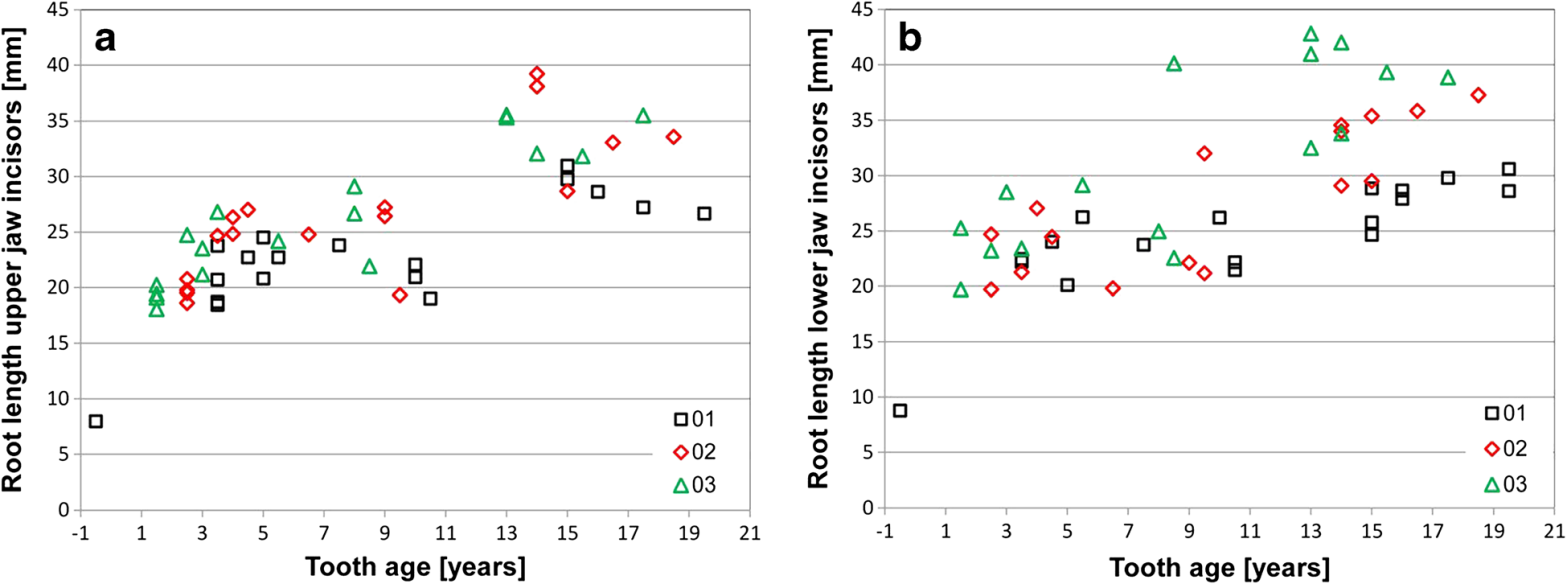
**Boxplots representing the increase of the dental root lentgh in time. a**: upper jaw incisors; **b**: lower jaw incisors 01 = center, 02 = middle, 03 = corner incisors; symbols indicate individual measurements.

**Figure 6:**
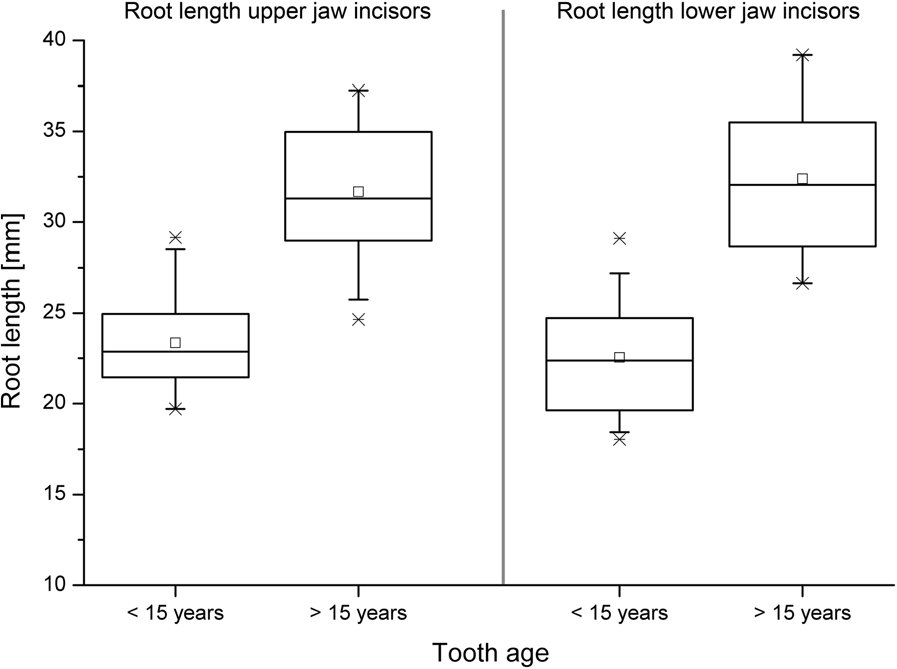
**Boxplots of the dental root length of erupted upper and lower jaw incisors.** Tooth age assigned to the root length. Box: interquartile range (25%, 75%), horizontal line: median, center point: mean, whiskers: range (5%, 95%), stars: minimum and maximum. The dental root length increases in time. Teeth older than 15 years p.e. show a significant longer root than younger teeth (p = 5.4 × 10^-13^ upper jaw, p = 2.4 × 10^-12^ lower jaw).

Due to the non-uniform shape of the enamel cover, a clear spatial distinction between dental crown (composed out of enamel, dentin and cementum) and dental root (composed of dentin and cementum only) appears difficult. Therefore the mean root length was calculated using the mean lengths of the tooth and the mean length of the enamel cover (as explained above). With advancing age the dental root increases in length. The data suggests that the dental root grows in staggered phases. Up to a tooth age of 10 to 15 years, a moderat increase in the root length can be seen. In incisors older than 10 years p.e., and more obvious in teeth older than 15 years p.e., dental roots are markedly elongated compared to the younger teeth (Figures 5 and 6).

Data concerning the relative length of the dental root (as a percentage of the total length of the tooth) is presented in Table 3. Incisors up to an age of about 15 years show a quite steady relative root length of 31 to 41%. Teeth older than 15 years p.e. show roots, which occupy 55 to 60% of the total length of the tooth.

**Table 3:** Root length as percentage of the tooth length

	**Center incisors**			**Middle incisors**			**Corner incisors**		
Tooth age (years p.e.)	< 10	10 -15	> 15	< 10	10 -15	> 15	< 10	10 -15	> 15
Upper jaw	31%	36%	55%	34%	38%	56%	36%	41%	60%
Lower jaw	32%	38%	52%	35%	35%	56%	37%	37%	60%

## Discussion

The performed μCT scans produced 2D images of high anatomical accuracy which allowed a reliable visualization of the dental substances in 3D models of equine teeth [13]. The computerized 3D models were suitable to obtain novel morphometric data of equine incisors. For the first time detailed measurements as well as three-dimensional analyses of different tooth substances and structures (infundibulum, crown, root) became possible. Unfortunately, μCT investigations are not feasible in the living horse due to technical limitations. Therefore, the presented study had to be designed as a cross sectional study obtaining morphometric measurements only at a single point in time from each tooth, which inevitably complicates data analysis and interpretation. However, the morphometric data of 103 incisors suggest typical morphologic and morphodynamic characteristics after statistical analyses.

### Maintained length of equine incisors

Equine incisors, pertaining to the high crowned hypsodont teeth, are subjected to continuous dental wear. The loss of dental hard substances at the occlusal surface is compensated by tooth growth [14]. Therefore dental material is added at the apical end of the tooth. Simultaneously, occlusion is maintained by tooth eruption achieved by the periodontal ligament. The separate processes of apical tooth growth and occlusal wear result in changes in the total length of the tooth when not evenly balanced [3]. According to our data, tooth growth exceeds dental wear for two to four years post eruption resulting in an increasing total length of the tooth. After that time, the total length of the tooth remains constant up to a tooth age of 13 to 15 years. In this period of time, tooth growth and dental wear appear to be well balanced. Hitherto, it is largely unknown how this balance is regulated. An attractive hypothesis comes from studies that demonstrated the perception of mechanical stimuli by human periodontal cells [15,16]. According to these data, mechanical loads activate several signal transducing molecules in periodontal cells. Subsequently cellular processes are initiated maintaining periodontal integrity [15,16]. Similar mechanisms of mechanotransduction might be also valid for the structures responsible for tooth growth, i.e. a long lasting equine enamel organ and equine epithelial root sheath. Accordingly a perception and translation of mechanical stimuli by odontogenic cells could explain the balanced processes of dental wear and tooth growth. At least the ability for mechanotransduction has been recently proposed for odontoblasts (cells producing dentin) [17].

### Post eruptive growth of equine incisors

The mere observation of post eruptive growth of equine incisors does not answer the question whether the tooth elongation is facilitated by root growth or by growth of the dental crown (i.e. prolonged enamel production). In previous studies crown formation (production of tooth with an enamel covering) and root formation (apical tooth formation without enamel production) has not been distinguished from each other and post eruptive tooth elongation was referred to as root formation [3,14]. Acquired data of this study permitted to estimate that the crown itself increases for a distinct period of time by post eruptive deposition of enamel. The length of the infundibula, when once formed cannot shorten except of being worn from the occlusal side. Thus, the distance between the apical infundibular limit and the apical edge of the enamel cover might serve as a useful mark to examine the post eruptive increases of the enamel cover. Unfortunately, such measurements would produce valid data only in a longitudinal study. In a cross sectional study presented here, the related data might be inaccurate, because the initial distance between the infundibular limit and the apical edge of the enamel cover might vary in a wide range between different horses and breeds. Nevertheless, the data obtained suggest a prolonged enamel production for several years after first eruption of the tooth. However, further histological investigations are needed to verify the existence of a productive enamel organ in equine incisors after eruption. For equine cheek teeth such a prolonged existence of the enamel organ for approximately five years after first tooth eruption has been already demonstrated [18,19].

Remarkably, in equine incisors there is no linear demarcation line between the dental crown (covered by enamel) and the dental root (composed of dentin and cementum). The non-uniform shape of the enamel cover displays mesial and distal indentations and therefore both an enamel organ and an epithelial root sheath exist at the same time in close proximity to each other in equine incisors. It is assumed that complete compensation of occlusal wear in incisors can only be accomplished as long as intact epithelial cell formations (enamel organ and epithelial root sheath) persist. The start of the decrease of tooth length with 13 to 15 years suggests a limited persistence of the epithelial formations up to this age. In incisors older than 15 years tooth elongation can only be facilitated by mere apposition of dental cementum and does not outbalance occlusal wear. This assumption is supported by the presented data concerning the relation of dental crown and dental root. In incisors with maintained length, up to an age of approximately 15 years, the percentage of the tooth covered by dental root increases, if at all, only moderately, which is a further indication of the existences of an active enamel organ in combination with a productive epithelial root sheath. In incisors displaying decreasing length, being older than 15 years, the relative length of the dental root increases markedly, indicating the absence of an enamel organ.

However, an alternative - or supplementary - explanation for a decreasing tooth length can be derived from observations of the infundibulum. According to own investigations (data not shown), the infundibulum disappears with 13–20 years. The tooth apical to the apical infundibular limit, that contains no longer infundibular enamel, may wear faster.

The timescale for the changes in incisor length given here are in between the scales which were suggested by studies of Van Foreest (1995) [14] and Muylle et al. (1999) [3]. Van Foreest [14] stated that teeth growth and root formation cease with 10–12 years. Muylle et al. [3] estimated that maximal tooth length was reached 2–3 years after eruption, maintaining until 17 years p.e. These similar but not identical results which were obtained in three different pools of specimens might indicate that large individual variations exist. However, despite the different timescales given in the literature, it has been generally accepted that incisor length remains constant for much more than ten years.

### Equine incisors and equine cheek teeth

It has been demonstrated that equine cheek teeth grow by enamel production, i.e. crown formation for approximately five years post eruption [18,19]. Thereafter, 5–15 years p.e., the epithelial root sheath exists and tooth elongation is facilitated by root formation, i.e. apposition of dentin and cementum. After 15 years, only apposition of dental cementum is observed [18,19]. Similar to our findings in equine incisors, the existence of the enamel organ in equine cheek teeth (i.e. approx. five years post eruption) seems to be correlated with the ability to fully compensate occlusal wear. Accordingly, it has been shown that equine cheek teeth preserve a constant length for approx. four years post eruption before occlusal wear overbalances the ability for tooth elongation [20]. These findings are further supported by various studies reporting that the cease of tooth elongation occurs in equine cheek teeth earlier than in incisors [20-23].

## Conclusions

The use of μCT images in this study, providing a resolution of less than 0.1 mm, enabled a detailed 3D description of the dental hard substances and allowed to estimate the timescales in which the incisor length remains stable. From a tooth age of 13 to 15 years post eruption the total length of the incisors decreases continuously. Consequently, the attachment area for the tooth supporting periodontal ligament decreases, resulting in increasing periodontal stress levels [24]. Thus, at an age of 13 to 15 years post eruption equine incisors are exposed to fundamental biomechanical changes within the periodontal ligament. These might be necessary but not sufficient preconditions for the onset of EOTRH, which is a typical disease of aged horses [12]. However, further biomechanical, cell biological and microbiological examinations are urgently needed to identify imperative factors for EOTRH.

## Abbreviations

PDL: Periodontal ligament; μCT: Micro-computed tomography; DICOM: Digital imaging and communications in medicine; ANOVA: Analysis of variance; p.e.: Post eruption.

## Competing interests

None of the authors has any financial or personal relationships which could inappropriately influence or bias the content of this paper.

## Authors’ contributions

PS designed the study, performed measurements, analyzed data, drafted and wrote the manuscript. ML contributed to the study design, data analysis and interpretation. HS contributed to the study design. CS contributed to the study design, data analysis and interpretation and helped with editing. All authors read and approved the final manuscript.

## Supplementary Material

Additional file 1 **Video of a 201 incisor (see Figure **4**a, b, c) showing enamel cover (white) and the pulp cavity (red).** Click here for file

Additional file 2 **Video of a 103 incisor (see Figure **4**d, e, f) showing enamel cover (white) and the pulp cavity (red).** Click here for file
